# Viruses-*to*-mobile genetic elements skew in the deep Atlantis II brine pool sediments

**DOI:** 10.1038/srep32704

**Published:** 2016-09-06

**Authors:** Mustafa Adel, Ali H. A. Elbehery, Sherry K. Aziz, Ramy K. Aziz, Hans-Peter Grossart, Rania Siam

**Affiliations:** 1 Biology Department, Biotechnology Graduate Program and YJ-Science and Technology Research Center, American University in Cairo, Egypt; 2 Department of Microbiology and Immunology, Faculty of Pharmacy, Cairo University, Egypt; 3 Leibniz-Institute of Freshwater Ecology and Inland Fisheries, Alte Fischerhütte 2, 16775 Stechlin, Germany; 4 Institute of Biochemistry and Biology, University of Potsdam, Am Neuen Palais 10, 14469 Potsdam, Germany

## Abstract

The central rift of the Red Sea has 25 brine pools with different physical and geochemical characteristics. Atlantis II (ATIID), Discovery Deeps (DD) and Chain Deep (CD) are characterized by high salinity, temperature and metal content. Several studies reported microbial communities in these brine pools, but few studies addressed the brine pool sediments. Therefore, sediment cores were collected from ATIID, DD, CD brine pools and an adjacent brine-influenced site. Sixteen different lithologic sediment sections were subjected to shotgun DNA pyrosequencing to generate 1.47 billion base pairs (1.47 × 10^9^ bp). We generated sediment-specific reads and attempted to annotate all reads. We report the phylogenetic and biochemical uniqueness of the deepest ATIID sulfur-rich brine pool sediments. In contrary to all other sediment sections, bacteria dominate the deepest ATIID sulfur-rich brine pool sediments. This decrease in virus-*to*-bacteria ratio in selected sections and depth coincided with an overrepresentation of mobile genetic elements. Skewing in the composition of viruses-*to*-mobile genetic elements may uniquely contribute to the distinct microbial consortium in sediments in proximity to hydrothermally active vents of the Red Sea and possibly in their surroundings, through differential horizontal gene transfer.

Tectonically induced deep, hypersaline, anoxic marine brine pools are found in the Red Sea, Mediterranean Sea and the Gulf of Mexico. The unique geochemical and physical characteristics of these topographic depressions were more studied than studies of biological life^1^.

The Red Sea rift contains 25 brine pools^2^,^3^,^4^. The rift is formed by the slow spreading motion of the African and Arabian plate, extending from north (Dead Sea rift) to south (Afar triple junction of the Aden ridge and East African rift)^3^,^5^. The origin of the Red Sea deeps and their brine pools is evident from the general structure of strata of the Red Sea bed. Evaporite strata, rich in minerals, were formed by tectonic and climatic changes that caused the desiccation of basins and seas^6^. Fissuring around the deep causes minerals to be leached by the invading deep water through the evaporite layer and hydrothermal activity may further influence brine formation^3^,^7^. Despite shared properties of Red Sea brine pools they are characterized by differential high heavy metal content and metalliferous sediments^1^. The Atlantis II area includes the largest brine pool Atlantis II (ATIID), Discovery Deep (DD) and Chain Deep (CD)^8^. Oxygen levels are low in ATIID and DD, the subjects of this study, measuring <1 ml/l below 2000 m depth, but Oxygen is fully absent at higher temperatures-in the ATIID lower brine layer^3^,^9^,^10^. Sources of light are scarce and limited to bioluminescence as the deeps are well beyond the photic zone^11^. Consequently, this deep oligotrophic extreme environment forms a niche of extremophilic organisms that are well adapted to such harsh conditions.

Both ATIID and DD brine pools have different geochemical and physical characteristics^3^. A slow rise in temperature over the last 50 years in ATIID was reported while DD remains fairly unchanged^3^,^12^. This high heat flux contributed to the ATIID brine pool richer mineral content, higher salinity and brine stratification^13^. The ATIID brine pool is the largest of all brines, covering an area of 55 km^2^. Its maximum depth is 2,170 m, and the accumulated metalliferous sediment varies in thickness from 10 to 30 m^3^,^4^,^14^. Its brine reaches up to 270 psu^13^, and it’s latest recorded temperature was 68 °C^9^,^10^. The DD brine pool is a smaller pool, covering 11.5 km^2^ with a maximum depth of 2,237 m^3^,^14^. The deep lies on the periphery of ATIID. The brine temperature was relatively low (48 °C)^9^,^10^. Metalliferous sediments in the deep brines have varying thickness and composition depending on the richness and precipitation of minerals from the overlying brine^9^. Chain Deep lacks hydrothermal activity and its temperature reaches up to 50.3 °C^8^.

Several culture-dependent and independent studies addressed microbial communities in the Red Sea brine pools, but only two studies addressed the selectively metal rich brine pool sediments^15^,^16^. The general microbial community in the ATIID sediment was similar to the overlying water column, as revealed by 16S rRNA and 454 shotgun sequencing^16^. A comparative 16S rRNA pyrotag analysis of sections from the ATIID and DD brine sediments identified unique microbial consortia in the deepest sulfur-rich ATIID sediment section and the deepest nitrogen-rich DD sediment section^15^. However, the microbial evolution in relation to differences in environmental conditions of the pool sediments remains to be explored. We hypothesize that lithologically different sections and the geochemical gradient will possess a complementary, genetically resistant microbial community to overcome the diversity of these extreme environments. Additionally, different horizontal genetic transfer mechanisms may have contributed to the evolution of the genetic makeup of microbes in this unique ecosystem. The lithologically different sediment sections, sampled from the 3.5m sediment core, had different depth. Seven Atlantis II sediment samples were studied (ATII-1;-6), the sample closest to the sea bed were samples ATII-1a and the deepest, ATII-6, was 3.4m from the sea-bed. Similarly Seven DD sediment samples were studied (DD-1;-7); DD-1 being the closest to the sea bed and the deepest, DD-7, was 3.4m from the sea-bed^15^. One sample from CD and another BI site were analyzed, both were a few centimeters from the seabed (Supplementary table 1).

To unveil such microbial communities we performed shotgun metagenomic sequencing and comprehensive analysis of 14 sediment sections of ATIID and DD, and two samples from CD and a Brine Influenced site (BI). A map illustrating the sampling sites in the Red Sea Atlantis II area is presented (Fig. 1). We aimed to identify sediment-specific reads for each sediment section. Through a custom *in-silico* approach and the classical homology-based alignment to reference sequences approach, we report the phylogenetic and metabolic potential uniqueness of the deepest ATIID brine pool sediments. Our results suggest a distinct microbial consortium, in proximity to hydrothermally active vents, which may have evolved through alternative horizontal genetic transfer mechanisms.

## Results

### Metagenomes from Red Sea ATIID, DD, CD and BI sediments

Out of ≈3.7 × 10^6^ reads (1.47 × 10^9^ bp) reads generated by shotgun sequencing of DNA from all sampled brine pools and brine-influenced sediment sites, ≈3.2 × 10^6^ reads (1.32 Gbp) were retained after quality control (QC) processing (Table 1 and Supplementary table 2a). None of the samples showed an appreciable change in GC content following QC (Supplementary table 2b).

Subtraction of ATIID water column and brine seawater reads from the sediment reads was performed to generate sediment specific reads. Atlantis II water column reads were used to subtract common reads from ATIID, DD, BI and CD sediment reads. This filtration step resulted in the utilization of 6.5 × 10^5^, 1 × 10^6^, 6.9 × 10^4^ and 8.1 × 10^4^ reads for ATIID, DD, BI and CD, respectively. The sediment specific reads, are presented for each sample/section (Supplementary table 3). Alpha rarefaction analysis of identified taxa in the sediment datasets, before or after subtraction of water column reads (Supplementary Figure 1), reached an asymptote in the diversity of the sediment-specific datasets compared to unfiltered datasets.

### Identification of sediment specific taxa in brine sediments

Domain level taxonomical classification (Fig. 2) showed bacterial related reads prevalence in the ATIID-1a and ATIID-1b datasets, contrary to all other ATIID, DD, BI or CD sections, which were dominated by viral related reads (Table 2). ATIID-1a and ATIID-1b had 32.7 and 20.09% bacterial reads compared to the remaining sediment samples that had 9.37% ± 0.29. Principal Component Analysis (PCA) of sediment-specific reads, based on species-level taxonomical classification, revealed the first three principal components accounted for 97.7% of the data variability, and the first principal component alone accounted for 91.5% of the variability. PCA showed that ATIID-1a and ATIID-1b sections were distant from all other sections, confirming their unique composition. All sections had a large proportion of no-match reads (68.86 ± 0.63% and 67.16 ± 0.94% in total un-subtracted and sediment specific datasets, respectively) and only 30% of the reads were annotated.

A distinct cluster of taxa, mostly bacteria, was identified in the deepest ATIID sections (Supplementary Figure 3). Out of 2,553 taxa identified, 684 sediment specific taxa showed a significant difference among sediment sections. Consequently, two-group comparison between ATIID-1a & 1b groups *vs.* all other sections, identified a distinct ATIID-1a & 1b cluster (Supplementary table 4). On the other hand, all sections differed from ATIID-1a & 1b mainly in the viral community.

The distinct ATIID-1a & 1b cluster includes: 1) Archaea: *Candidatus Parvarchaeum* (Euryarchaeota), *Nanoarchaeum equitans* (Nanoarchaeota), and *Candidatus Nitrosoarchaeum koreensis* (Thaumarchaeota), 2) Viruses: only ssDNA virus Nanoviridae and 3) diverse bacterial community taxa. These include a distinct consortium of Actinobacteria, Aquifica, Chloroflexi, Synechococcus, Acaryochloris, Firmicutes as well as α, β and γ- proteobacteria (Supplementary table 3).

The combination of PhAnToMe and GAAS analysis confirmed a dramatic decrease in abundance and diversity of known viruses in ATIID-1a&b sections. These deepest two layers of the Red Sea (ATIID-1) showed a low relative abundance of known viral sequences (1.19% and 2.35%, respectively) (Table 2). The ATIID subsections also demonstrated the lowest viral alpha-diversity, and their viral fraction was largely dominated by gokushoviruses (30.5% and 54.5% of total virome in ATIID-1a and -1b, respectively) followed by uncultured Mediterranean phages (52.3% and 24.8% of total virome in ATIID-1a and -1b, respectively). On the other hand, different Mediterranean phage types and Cyanophages were the most abundant in the remaining sediment sections (85.4% ± 0.3 and 4.8% ± 0.1 for each phage, respectively). Additionally, the virome analysis revealed the dominance of several Mediterranean phage-like viruses in the Red Sea sediment metagenome. The uppermost Discovery Deep section, closest to the seabed (DD-7) was the richest (1,662 different species) and most virally diverse section (Shannon diversity = 5.44).

### Selected sediment specific metabolic potential

PCA of functional assignments using SEED classification^17^ showed that the 16 sediment sections generated three principal components that account for 79.3% of the variability in the data, with the first PCA component describing 51.2% of the variance. Similar to the phylogenetic classification, ATIID-1a & 1b were quite distant from other sediment samples (Supplementary Figure 4a). Repeating the analysis with KEGG orthologous group classification^18^ generated similar results (Supplementary Figure 4b). As expected, diverse SEED subsystems involved in several metabolic pathways were detected. Most relevant were Sulfur, nucleotide metabolism and DNA replication and repair, which seem to be responsible for distinguishing ATIID-1a & 1b from the rest of the sediment sections (Fig. 3). Sulfur metabolism pathway in these ATIID-1a & 1b sections represented 0.38 ± 0.01% of the total metagenomic reads, a fraction that is significantly higher, compared to other sections (Fig. 3a).

A two-group comparison between the sediment specific and total unfiltered datasets, based on SEED subsystems and KEGG functional classifications, allowed the identification of functions likely to be unique to the sediment. Arylsulfatase, aryl-sulfate sulfohydrolase, involved in organic sulfur assimilation and internalin-like proteins involved in virulence were unique to the sediment specific samples. The same comparison using KEGG classification identified homologues to the K05747 orthologous group (Wiskott-Aldrich syndrome protein) involved in cell motility (Supplementary Figure 5).

### Abundance and diversity of mobile genetic elements

We analyzed three different mobile genetic elements: plasmids, integrons and insertion sequences (*IS*). Interestingly, ATIID-1a&b showed the highest abundance and diversity of the three mobile elements in almost all cases (Fig. 4a,b). In general, *IS* were more abundant than integrons and plasmids. The lowest *IS* abundance was detected in sections 1&7 of DD. Integrons could not be detected in 10 out of 16 (62.5%) different lithologic sections (ATIID-3,4,5&7; DD-1,2,3&6; CD and BI). The lowest abundance of plasmids was found in BI. All detected *IS* were classified into their corresponding families, and *IS* family abundance was computed for each dataset (Fig. 4c). Based on the level and types of *IS* families, ATIID-1a and b showed a unique cluster. The correlation coefficient (r) and P-value illustrate a positive correlation between bacteria and MGEs abundance and a negative correlation between viruses and MGEs abundance (Supplementary table 6).

## Discussion

### A Red Sea brine pool sediment metagenomic study

This study was launched to enrich our knowledge about the biological diversity of the Red Sea brine pool sediments, which are understudied compared to the water column. A challenge to any marine metagenomic study is ruling out contamination with overlying water column, and to address this we computationally eliminated reads in common with the ATIID water column and overlying brine and generated sediment-specific reads (Supplementary table 3). This filtration step was justified by a previous study demonstrating no apparent differences in community structure at the same depth in water from the ATIID and the DD sites of the Red Sea^19^. These eliminated reads are not necessarily contamination of overlying water column, but may represent shared communities with the sediment, e.g. due to a frequent exchange. This study exclusively focused on sediment specific microbes and metabolic potential.

### Unique microbial structure and metabolic potential in Red Sea sediments

Comparison of phylogeny data generated from the current study to the previous 16S rRNA taxa assignment^15^ revealed 10-folds increase in the microbial richness (Supplementary table 5). This could be due to the amplification bias generated from 16S V6V4 rRNA pyrotags^20^. Approximately 30% of the sediment-specific reads were well annotated reads and the remaining 70% of the reads had no match to the current database (Fig. 2). Many of these annotated sediment-specific reads were homologous to genes of dsDNA phages: myoviruses, siphoviruses and podoviruses, all of which were homologous to phages infecting cyanobacteria of the *Synechococcus* and *Prochlorococcus* genera, which inhabit the photic zone of surface water^21^,^22^. The reason that these reads were retained even after the filtrations step may be due to the modular nature of these viruses and their broad host range^21^,^22^, therefore escaping elimination. Alternatively if such reads were generated from free phages, and not prophage, then these viruses might have originally stemmed from the overlying water column and sunk down but evolved, over time, in these extreme environments.

To identify sediment-specific metabolic potential, we compared functional classifications of sediment-specific and total unfiltered reads. This included selected sulfur assimilation (Arylsulfatase), cell motility (“Wiskott-Aldrich syndrome protein”) and virulence processes (internalin-like proteins) (Supplementary Figure 5). The identification of internalin-like proteins to be unique to the sediment specific samples is in agreement with the role of viral infection and suggests that these deep marine sediments are hotspots of viral abundance and potentially of viral infection. Reads assigned to DNA repair and nucleotide metabolism, were enriched in sections other than ATIID-1a and b, this could be due to the observed prevalence of viruses, and the new requirement imposed on the infected hosts (Fig. 3).

### Marked skew in viruses-*to*-prokaryote-*to*-Mobile Genetic Elements ratio in sulfur-rich deep marine sediment sections

Our previous study revealed a relatively high total sulfur content in ATIID-1a & 1b, apparent from the CHNS profiling^15^. Comparison of the S-rich ATII-1 sections with other sections revealed several abundant taxa and proteins known to be involved in sulfur metabolism (Fig. 3, Supplementary table 5). This finding further suggests that sulfur metabolism shapes the microbial community structure^15^,^23^, despite the fact that this pathway was less prevalent than other microbial metabolic potential identified in the metagenome.

The prevalence of viruses in all sediment sections, with the exception of the S-rich ATIID-1a & 1b sections, has been previously reported in the deep sea and is suggested to recycle biomass and maintain diversity of the microbial community^22^,^24^,^25^. We suggest that the higher virus-*to*-bacteria ratio suggests a higher virus-*to*-host ratio and a higher rates of lysogenic *vs.* lytic infection in these habitats^26^, however more data need to be generated on the host abundance and diversity in these sediments.

Comparative metagenomic analysis showed the deeper ATIID-1a & 1b sediment layers to be distinct from the remaining sediment sections. ATIID-1a & 1b sediment microbial communities were comprised of mainly bacterial taxa. Mobile genetic elements (MGE) were overrepresented in the ATIID-1a & 1b metagenomes including plasmids, insertion sequences and integrons (Fig. 4a,b). Under selected environmental stresses a competitive role may be implied between MGEs and viruses, because of the inverse correlation observed and that the environmental stress in ATIID-1a & 1b sections favors the role of MGEs in horizontal gene transfer and microbial evolution.

This study is an environmental genomics study of three Red Sea brine pool sediments and an adjacent brine-influenced site. The different sampling locations and the lithologically dissected sediment cores downstream analysis revealed an accurate precision of microbial stratification derived from a similarly accurate precision in evolutionary mechanism. The skew of the virus-*to*-bacteria ratio in only the deeper, sulfur-rich samples, not common in other deep-sea marine systems including hydrothermal vents, suggests an alternative horizontal gene transfer mechanism that contribute largely to the evolution of microbes in the specified narrow section of deep sea sediments. The skew in the viruses-*to*-mobile genetic elements ratio in only these deeper and sulfur-rich samples suggests that mobile genetic elements contribute largely to the evolution of microbes at these sites. Further studies should examine the role of viruses-*to*-mobile genetic elements ratio in these selected deep-sea habitats. Additionally, habitat preferences for the prevalence of such alternative horizontal transfer mechanism need to be explored.

## Methods

### Sampling and generation of metagenomic datasets

**Figure 1:**
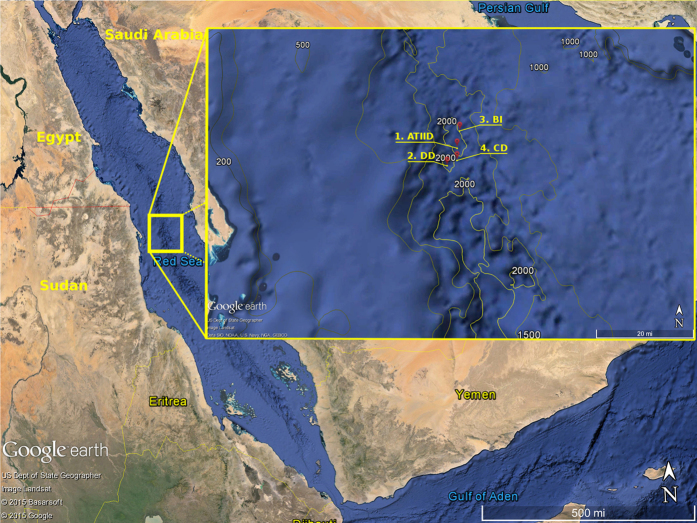
Sampling sites of Red Sea Atlantis II area. Samples were collected during KAUST Red Sea Expedition on April 2010. Sampling locations of the 1-Atlantis II (ATII), 2-Discovery Deep (DD), 3-Chain Deep (CD) and 4-the brine-influenced (BI) sites are presented. The figure was generated using Google Earth v 7.1.4.1529 © 2015 Google and data provided to the software by Basarsoft, US Dept. of Sate Geographer, Image Landsat, Data SIO, NOAA, U.S. Navy, NGA GEBCO. The overlaid bathymetric contours © 2014, GRID-Arendal.

**Figure 2:**
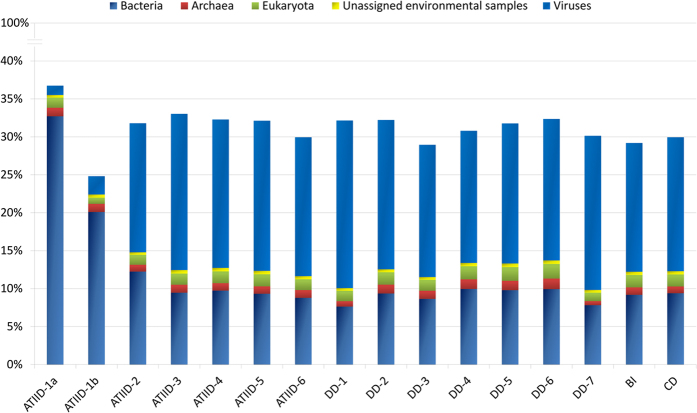
Domain distribution of metagenomic reads in ATIID, DD, CD and BI sediment sections. Classification of the metagenomic reads at the domain level, following elimination of water column reads. The bar graph shows the prevalence reads of bacterial, viral and archaeal origin. Unknown reads for each sample (not illustrated) represent the rest of the bar up to 100%. The classified bacterial community in ATIID-1a and ATIID-1b sections is high compared to the remaining samples. Reads of viral origin are higher in the remaining ATIID, DD, CD and BI samples. The figure was generated by MEGAN software v. 4.70.4 with modifications.

**Figure 3:**
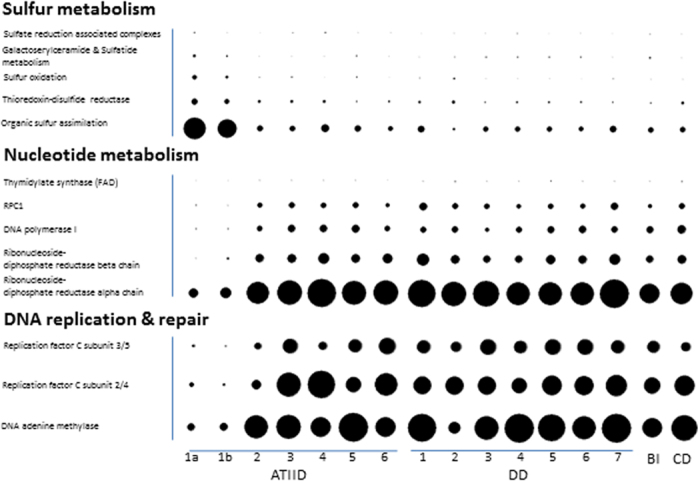
Sulfur metabolism, nucleotides metabolism, DNA replication and repair in all sediment metagenomic reads. Visualization of (**a**) Sulfur metabolism utilizing the SEED classification and (**b**) Differentially represented KEGG orthologous groups in nucleotides metabolism, DNA replication and repair categories. Frequencies are normalized to total identified reads per sample. The figure was generated by MEGAN software v. 4.70.4 with modifications.

**Figure 4:**
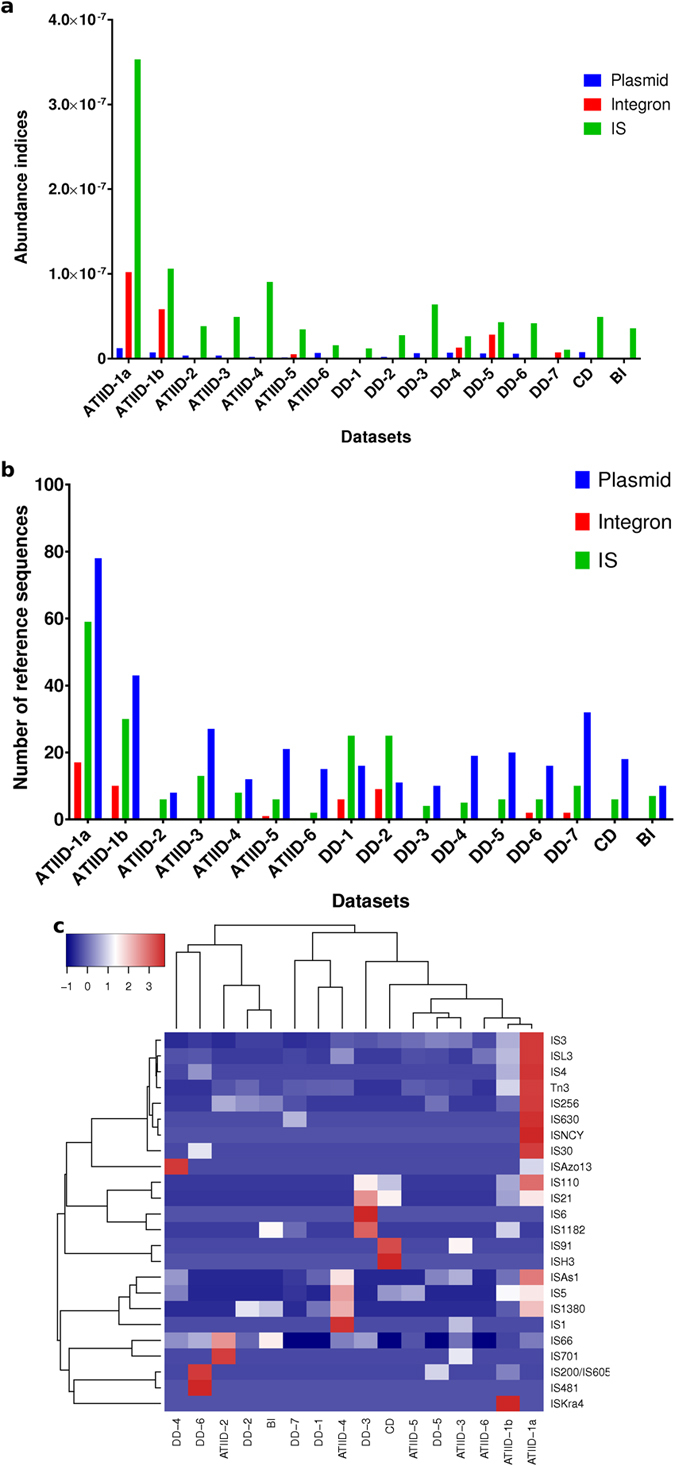
Abundance and diversity of mobile genetic elements in the Red Sea metagenomes. (**a**) Abundance of mobile genetic elements. (**b**) Diversity of mobile genetic elements. (**c**) Heat map based on the abundance of insertion sequence families. ATIID, Atlantis II Deep; DD, Discovery Deep; CD; Chain Deep, BI, Brine Influenced; *IS*, Insertion sequences. Charts (**a,b**) were generated by GraphPad Prism software, while heatmap (**c**) was generated by heatmap package in R environment.

Sampling was performed in spring 2010, on board the Hellenic Center for Marine Research (HCMR) research vessel Aegaeo^15^. Locations of the sampling sites are presented in Fig. 1. The 3.5 m sediment core, was used to sample lithologically distinct seven Atlantis II and seven Discovery Deep samples. DD-1 and ATII-1a, were the deepest samples, ATII-1b was a few centimeters away, but was lithologically distinct. The sediment samples, closest to the seabed, were ATII-6 and DD-7, at 3.4 m deep from the sea-bed. Only one sample was isolated from CD and BI sites, and both were a few centimeters away from the seabed^15^ (Supplementary table 1). Sampling equipment, physical and CHNS profile of the four sites, the two brine pools ATIID, DD, CD and BI sites are presented in supplementary table 2 and were previously reported^15^. DNA was extracted from each sample. 0.5–1 g with PowerSoil^®^ DNA isolation kit (MO-BIO, Calsbad, CA) at the American University in Cairo (AUC)^27^. 1 μg of DNA, from each sample, was subjected to direct metagenomic DNA 454 shotgun pyrosequencing library was constructed as recommended by GS FLX Roche Titanium guide, and the Double SPRI-TE Method (Beckman Coulter) was used for nucleic acid extraction via magnetic bead DNA fragment size selection. The libraries were sequenced with 454 GS FLX Titanium technology (454 Life Sciences) at the AUC laboratory.

### Bioinformatic analyses

#### Raw metagenomic datasets quality control (QC) processing

Metagenomic datasets in raw sequence flow format from 454 GS FLX/FLX+ Data Processing Software were exported and pre-processed using the PRINSEQ software^28^,^29^. Read ends with base quality value <15 (corresponding to about ≈97% base calling accuracy) and sequences of length below 60 bp were trimmed. The low complexity filter was used to exclude sequences having entropy <50. An overall quality filter was applied, allowing for a mean quality score of ≥15 along the whole trimmed sequence length. Sequences with ambiguity characters, >5% of their entire length, were removed. Finally exact and near identical (down to 98% similarity) 454 artificial replicates “ghost reads sequencing artifact”^30^ were removed using CD-HIT-454 software^31^ default criteria.

#### Generation of sediment-specific reads

QC reads from the 16 sediment datasets were pooled, followed by mpiBLASTn^32^,^33^ alignment with reads from datasets of overlying water column and brine^34^. ATIID brine/water samples were subtracted from all sediment reads, additionally ATIID and DD brine reads were subtracted from ATIID and DD sediment section datasets, respectively. Default criteria with soft masking enabled, were used in alignment, and reads from each section were filtered out if they had a length of ≥40 bp and a minimum identity of 90% to the reads from any of its target water or brine datasets were filtered out.

Further virome analyses utilizing both GAAS^35^ and PhAnToMe (*PHage ANnotation TOols and MEthods)* - http://www.phantome.org/Downloads/36 as performed to estimate the taxonomic composition and average genome size of phage-like elements in the metagenomic samples. With GAAS, the filtering criteria used an E-value threshold of (1 × 10e^−6^) and a minimum alignment similarity of 75%. The PhAnToMe database used was downloaded on September 1, 2015.

#### Protein-based phylogeny and function profiling of metagenomic datasets

The sediment reads were annotated by mpiBLASTx^32^,^33^ alignment to NCBI nr protein database (Jan. 2013). Default search criteria and soft filtering were enabled and a maximum of 100 hits per query read were used by the MEGAN^37^ software lowest common ancestor classifier algorithm. Additionally, built-in RefSeq mapping of NCBI nr to SEED^17^ and KEGG databases^18^ was used to generate function profiles. The generated profiles for the individual 16 sections were combined and used to build frequency read counts for downstream comparisons. Initially, we filtered frequency data to features with at least one statistically significant difference between any two datasets, using multiple pairwise two-tailed Fisher’s Exact Tests (FET)^38^,^39^ and a sequential Bonferroni adjusted p-value threshold of 0.05. Each of the phylogenetic, SEED and KEGG classifications were subjected to such filtering using a custom script that utilized PERL interface to R^40^ base functions. Differences between datasets were visualized as heat maps of log_2_(x + 1)-transformed, standardized to z-scores, and hierarchically clustered data. The z-scores were uniformly scaled from 0 to 1. The heat maps were generated using the “enhanced heat map function” from the R package gplots^41^. Two-way complete linkage hierarchical clustering was performed with 1,000 bootstraps by pvclust R package^42^ modified to use a Spearman’s correlation matrix generated from the standardized z-scores.

In addition, different datasets were compared based on phylogenetic, SEED and KEGG classifications. Statistical significant differences were identified and visualized using the Statistical Analysis of Metagenomic Profiles (STAMP) software^43^. The following two-group comparisons were considered: the total unfiltered datasets *vs.* sediment specific ones, and ATIID-1 sections *vs.* all other brine pool sediment sections. The two-group comparisons used White’s non-parametric t-test^44^ for detecting significant differences in mean relative proportions and were set to 10,000 permutations with Storey’s false discovery rate correction^45^ and a 0.05 p-value cut-off. Comparisons between total datasets and the corresponding sediment specific datasets were further investigated using the two sample comparison Fisher’s Exact Test^38^,^39^ and the more conservative Bonferroni correction with a 0.05 p-value cut-off.

#### Identifying Mobile Genetic Element reads

Abundance and diversity of plasmids, insertion sequences (IS) and integrons were assessed by matching the metagenomic data to the RefSeq database (ftp://ftp.ncbi.nlm.nih.gov/refseq/release/plasmid/, downloaded on January 26, 2014), INTEGRALL database^46^ (http://integrall.bio.ua.pt/, downloaded on January 29, 2014) and IS-Finder database^47^ (https://www-is.biotoul.fr/, downloaded on July 9, 2014). BLASTN was used to align sequence reads to the former databases using BLASTn (E-value threshold = 10^−5^). A read was annotated as a plasmid-like read if it aligned with >95% identity, over at least 90 nucleotides^48^. On the other hand, a read was assigned to either integrons or IS if it aligned with members of the respective database over 50 nucleotides with more than 90% identity^48^,^49^. MGE reads were normalized to calculate plasmid abundance index (PAI), abundance index (IAI) and IS abundance index (ISAI).


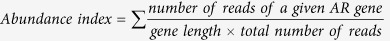


All the samples in this study were submitted to NCBI SRA under bio-project accession PRJNA299097 - SRP064947 titled “Metagenomes of Sediments from Red Sea Atlantis II, Discovery and Chain Deep Brine Pools”.

## Additional Information

**How to cite this article**: Adel, M. *et al*. Viruses-*to*-mobile genetic elements skew in the deep Atlantis II brine pool sediments. *Sci. Rep.* **6**, 32704; doi: 10.1038/srep32704 (2016).

## Supplementary Material

Supplementary Information

## Figures and Tables

**Table 1:** Metagenome generated for each sample dataset.

Sediment section	Raw bases	QC bases	% change	Raw reads	QC reads	% change
ATIID-1a	1.88E + 08	1.73E + 08	−7.7	428867	388642	−9.38
ATIID-1b	1.86E + 08	1.72E + 08	−7.68	430082	390240	−9.26
ATIID-2	41798945	32094845	−23.22	112217	81388	−27.47
ATIID-3	92830810	87570672	−5.67	225998	209973	−7.09
ATIID-4	24337335	22436407	−7.81	64363	57447	−10.75
ATIID-5	54322766	50947554	−6.21	136276	124488	−8.65
ATIID-6	33382989	29905377	−10.42	101410	88058	−13.17
DD-1	95398909	89705574	−5.97	261342	240352	−8.03
DD-2	61152953	43771566	−28.42	153548	101602	−33.83
DD-3	30783147	24703095	−19.75	87348	66033	−24.4
DD-4	66956263	50811630	−24.11	180714	129184	−28.51
DD-5	81859700	73776000	−9.88	177953	157017	−11.76
DD-6	96080706	90133197	−6.19	197343	182942	−7.3
DD-7	2.97E + 08	2.7E + 08	−9.02	913497	780210	−14.59
BI	56795456	52793284	−7.05	113930	104043	−8.68
CD	65473367	59792448	−8.68	139638	124159	−11.09

Total number of bps/reads generated for each sample, the remaining bp/reads following Quality Control (QC) process and the percent change in total number following QC processing.

**Table 2:** Virome abundance and diversity in Red Sea sediments.

Sediment section	Virome abundance	Shannon Diversity index
ATIID-1a	2.11%	4.082446
ATIID-1b	3.54%	4.23646
ATIID-2	20.47%	5.346457
ATIID-3	22.80%	5.34267
ATIID-4	23.86%	5.291893
ATIID-5	22.84%	5.357928
ATIID-6	21.73%	5.389315
DD-1	23.85%	5.26425
DD-2	23.15%	5.32654
DD-3	21.68%	5.203912
DD-4	20.44%	5.385041
DD-5	21.48%	5.347178
DD-6	21.32%	5.255225
DD-7	21.12%	5.441781
BI	20.30%	5.342897
CD	20.86%	5.364535

The percentage of viral reads in each metagenome sediment section (virome abundance) and Shannon Diversity indices of the viromes are presented.
