# Supplementary material for: Viruses-to-mobile genetic elements skew in the deep Atlantis II brine pool sediments
Source: Sci Rep. 2016 Sep 6;6:32704. doi: 10.1038/srep32704 (PMC5011723; doi:10.1038/srep32704)
Supplement: Supplementary Information [file srep32704-s1.pdf]

***Viruses-to-mobile genetic elements* skew in the deep Atlantis II brine pool sediments**

Mustafa Adel, Ali H. A. Elbehery, Sherry K. Aziz, Ramy K. Aziz, Hans-Peter Grossart and Rania Siam

Supplemental Figure 1

a.

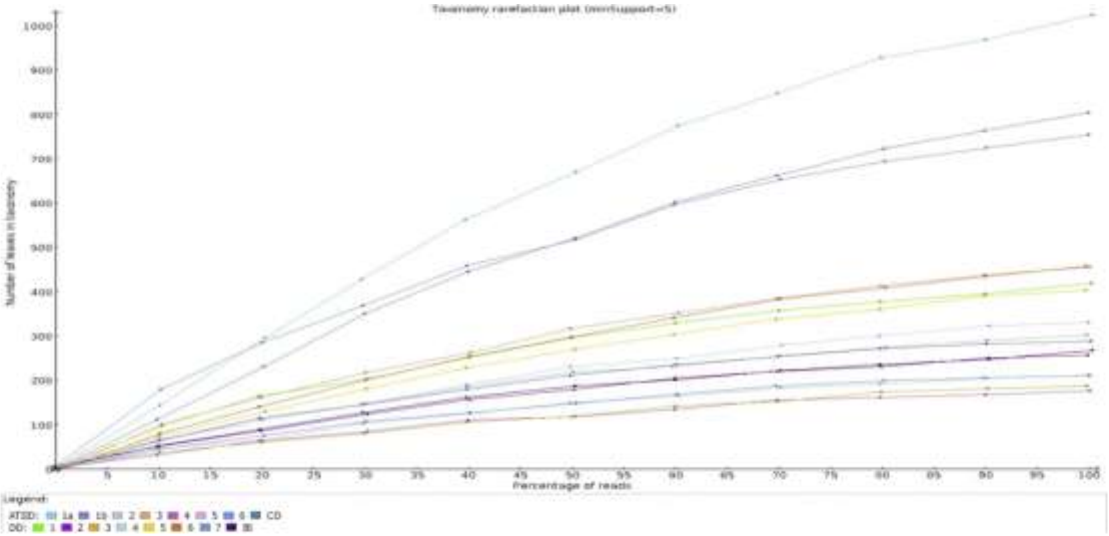

b.

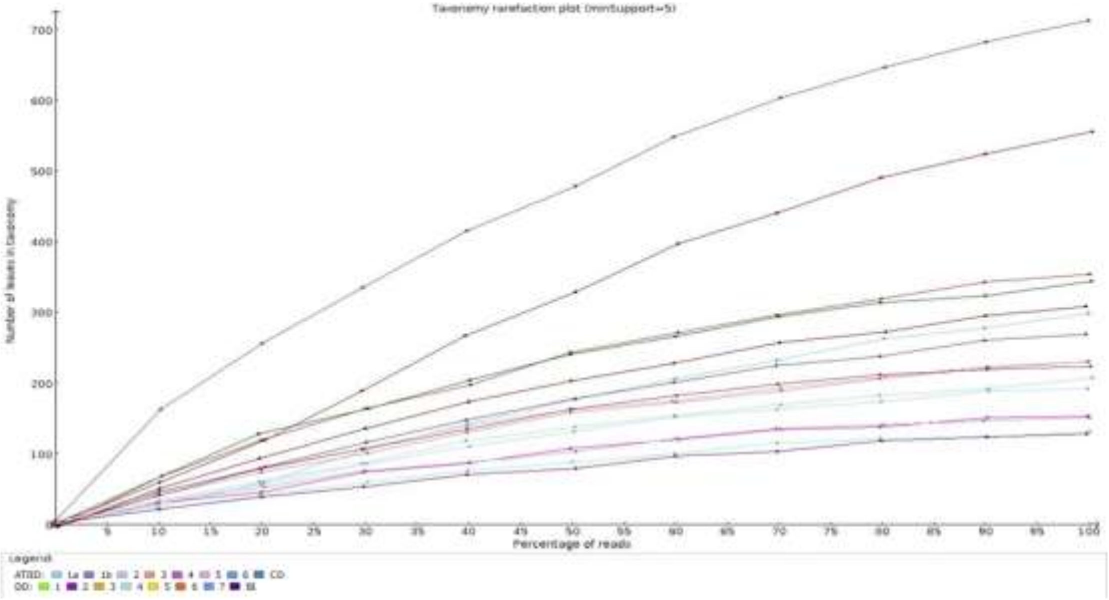

Supplementary Figure 1: Alpha rarefaction plots for identified taxa in different sediment samples sections
a) pre- and **b**) post-subtraction (sediment specific) process. Taxonomical classification is performed to Lowest Common Ancestor by MEGAN.

Supplemental Figure 2

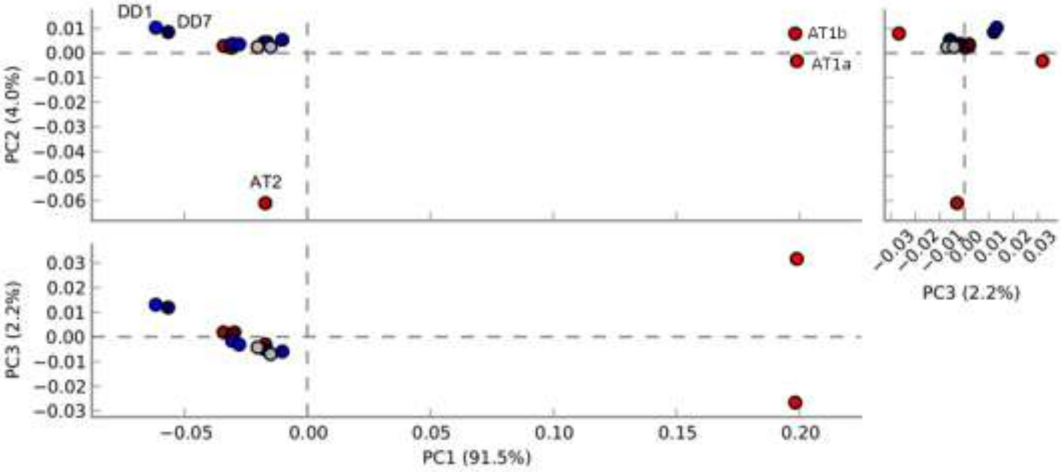

Supplementary Figure 2: Principal Component Analysis (PCA) of sediment samples PCA was based on taxonomical classification down to species level, and frequencies normalized to totals. ATIID sections are colored in increasing shades of red, DD similarly in blue, while CD and BI sites in grey.

Supplemental Figure 3

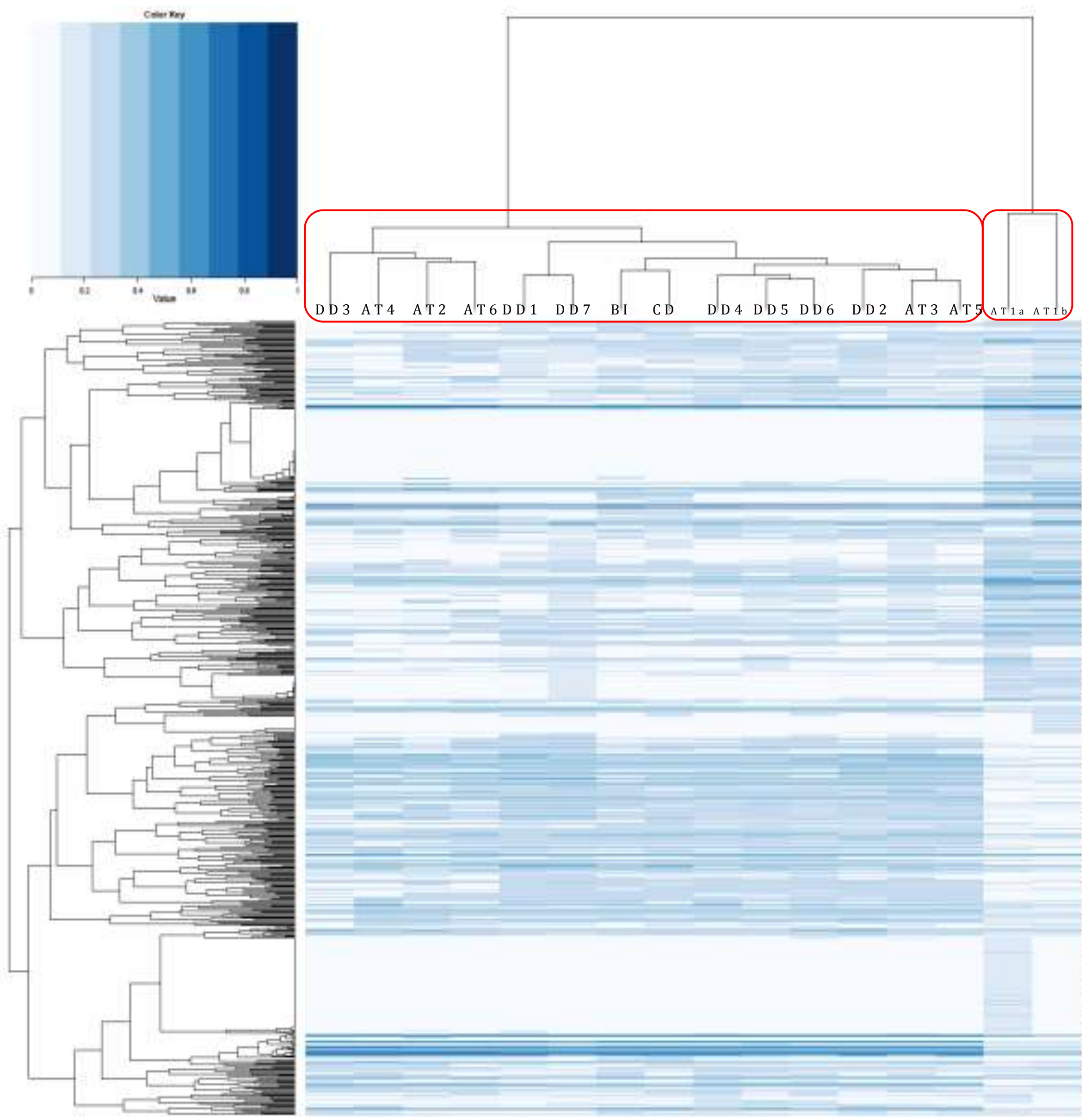

Supplementary Figure 3: Heatmap representation of hierarchical clustering of sediment specific microbial communities in the Red Sea sediments

Only significantly differing sediment specific microbial communities at the species level among sediment sections are presented. The bacterial community in the deepest two ATIID-1a and ATIID-1b sections is high compared to the remaining samples and responsible for the distinction into a group, while reads of viral origin are higher in the remaining sample sections.

Supplemental Figure 4

4.a

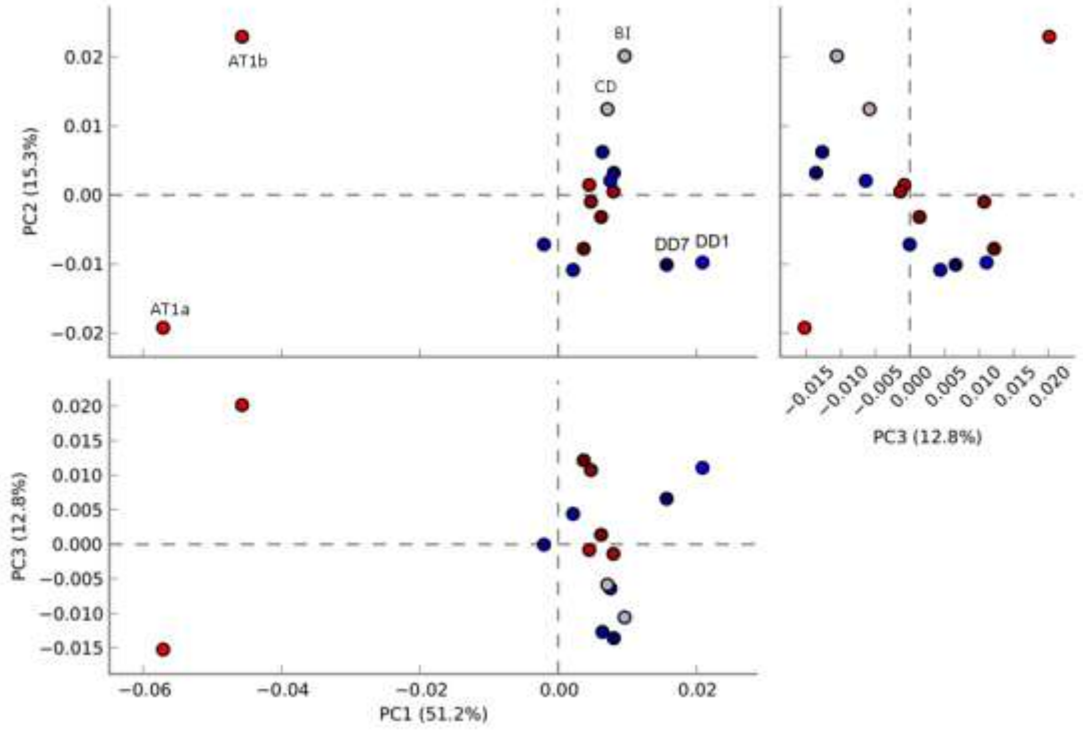

4.b

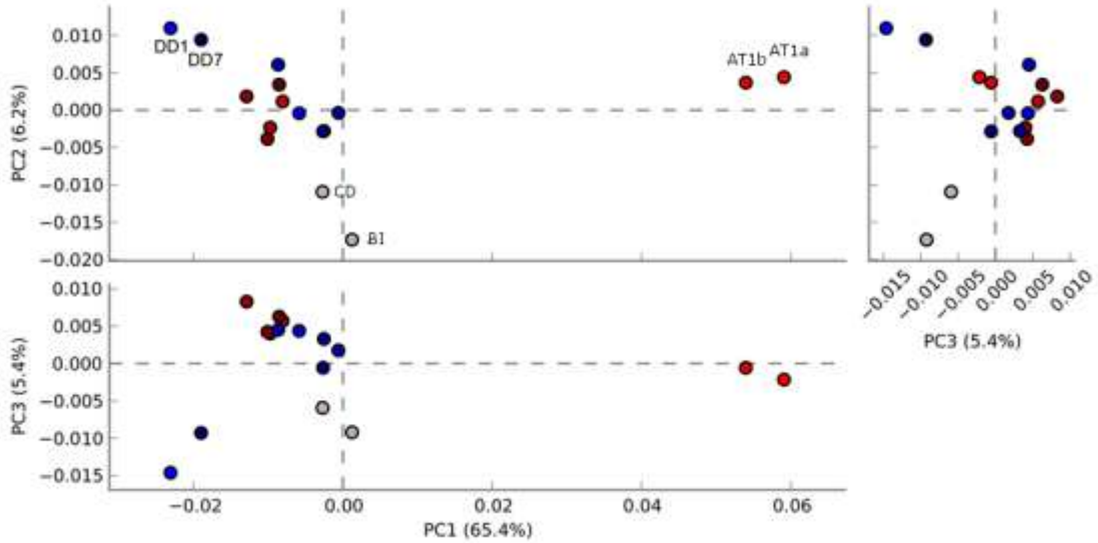

Supplementary Figure 4: **Principal Component Analysis (PCA) of sediment samples sections**

a) SEED classification. **b)** KEGG orthologous groups. Frequencies were normalized to totals. ATIID sections are colored in increasing shades of red, DD similarly in blue, while CD and BI sites in grey.

Supplemental Figure 5

a. Arylsulfatase

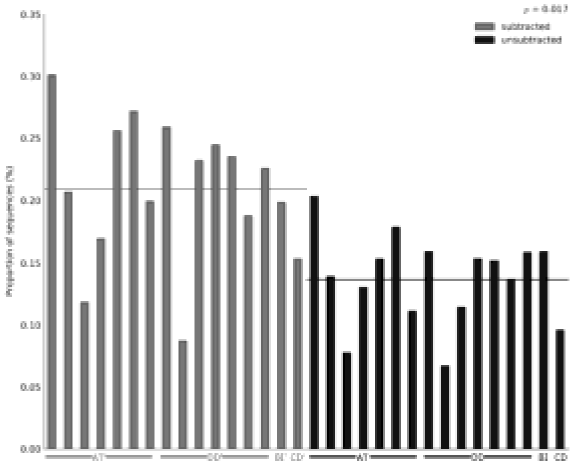

b. Internalin-like proteins

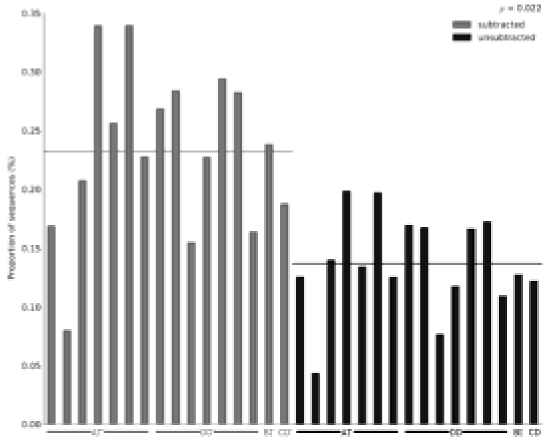

c. K05747 orthologous group "Wiskott-Aldrich syndrome protein"

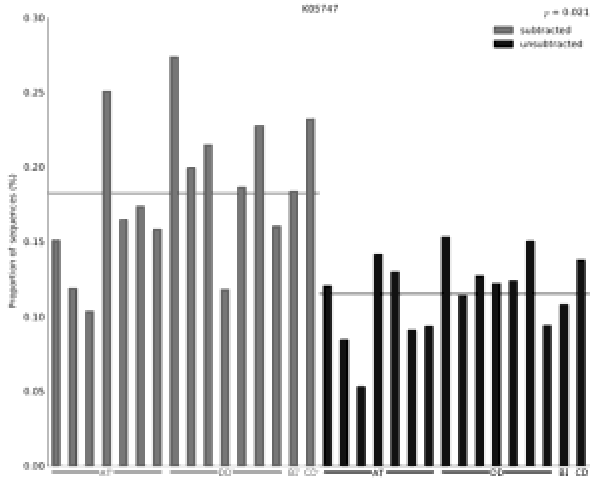

Supplementary Figure 5: **Proteins significantly detected in the Sediment Specific Reads**

Only proteins that showed significant increase of mean relative frequency in the subtracted (sediment specific) group of datasets ($P < 0.05$), as compared to the un-subtracted datasets, are shown.

Supplementary Table 1 – Metadata for the sampled subseafloor sediments

Location Name	Collection date	Depth (m)	lat.-lon.	temp. (°C)	Section Title	Position Below Seabed (m)
Red Sea, Atlantis II Deep	26/04/2010	2168	21.3455333 N 38.0839833 E	68	ATIID-1a	3.34
					ATIID-1b	3
					ATIID-2	2.7
					ATIID-3	1.43
					ATIID-4	1.15
					ATIID-5	0.5
					ATIID-6	0.15
Red Sea, Discovery Deep	26/04/2010	2180	21.28475 N 38.0484 E	45	DD-1	3.34
					DD-2	3
					DD-3	2.5
					DD-4	2
					DD-5	1.3
					DD-6	0.5
					DD-7	0.15
Red Sea, Chain Deep	27/04/2010	1937	21.3025833 N 38.0834 E	32	CD	0.22
Red Sea, Brine Influenced site	21/04/2010	1856	21.4088667 N 38.0937167 E	22	BI	0.05

Supplementary Table 2:

a) The mean length of reads in each sample and the effect of Quality control (QC) on length distribution.

Length Distribution

	Mean (bp)		Mode (bp)		Range (bp)	
**Sample**	**Raw**	**QC**	**Raw**	**QC**	**Raw**	**QC**
**ATIID-1a**	437.38 ± 156.80	445.48 ± 148.87	505	505	40 : 1123	60 : 902
**ATIID-1b**	433.14 ± 147.34	440.70 ± 139.85	513	507	40 : 865	60 : 811
**ATIID-2**	372.48 ± 143.59	394.34 ± 134.48	433	434	40 : 1281	60 : 881
**ATIID-3**	410.76 ± 132.17	417.06 ± 126.06	477	447	40 : 1594	60 : 910
**ATIID-4**	378.13 ± 141.01	390.56 ± 130.56	459	485	40 : 1329	60 : 788
**ATIID-5**	398.62 ± 133.53	409.26 ± 122.91	464	447	40 : 1289	60 : 811
**ATIID-6**	329.19 ± 128.68	339.61 ± 122.81	371	385	40 : 1596	60 : 728
**DD-1**	365.03 ± 145.71	373.23 ± 138.42	498	498	40 : 1204	60 : 683
**DD-2**	398.27 ± 144.51	430.81 ± 134.15	462	503	40 : 1248	60 : 784
**DD-3**	352.42 ± 149.00	374.10 ± 142.06	433	458	40 : 1164	60 : 768
**DD-4**	370.51 ± 136.59	393.33 ± 126.60	441	447	40 : 1290	60 : 742
**DD-5**	460.01 ± 152.99	469.86 ± 143.70	533	587	40 : 1596	60 : 890
**DD-6**	486.87 ± 151.71	492.69 ± 143.28	582	634	40 : 1478	60 : 968
**DD-7**	325.29 ± 166.65	346.50 ± 158.88	52	61	40 : 1594	60 : 880
**BI**	498.51 ± 155.54	507.42 ± 144.10	586	634	40 : 1595	60 : 886
**CD**	468.88 ± 155.85	481.58 ± 142.89	543	634	40 : 1186	60 : 1177

b) The GC content distribution per dataset before/after Quality control (QC).

GC Content Distribution

	Mean (%)		Mode (%)		Range (%)	
**Sample**	**Raw**	**QC**	**Raw**	**QC**	**Raw**	**QC**
**ATIID-1a**	44.31 ± 9.65	44.28 ± 9.63	41	41	0 : 95	7 : 95
**ATIID-1b**	41.90 ± 8.60	41.85 ± 8.58	40	40	4 : 82	7 : 81
**ATIID-2**	42.32 ± 9.78	39.57 ± 8.96	36	35	2 : 85	9 : 84
**ATIID-3**	37.24 ± 7.81	37.20 ± 7.74	35	35	1 : 86	1 : 83
**ATIID-4**	37.93 ± 8.51	37.75 ± 8.31	36	35	4 : 93	5 : 83
**ATIID-5**	37.43 ± 8.27	37.30 ± 8.12	35	35	3 : 87	7 : 87
**ATIID-6**	38.21 ± 8.82	38.11 ± 8.72	35	35	0 : 84	10 : 83
**DD-1**	37.09 ± 7.62	36.98 ± 7.51	35	35	0 : 85	7 : 85
**DD-2**	38.82 ± 8.95	37.93 ± 8.41	35	35	0 : 84	2 : 79
**DD-3**	39.00 ± 9.02	38.74 ± 8.85	36	36	4 : 85	6 : 85
**DD-4**	39.17 ± 9.13	38.93 ± 9.00	35	36	1 : 87	8 : 87
**DD-5**	38.58 ± 8.76	38.49 ± 8.67	35	36	0 : 84	6 : 80
**DD-6**	38.61 ± 8.76	38.54 ± 8.67	35	35	0 : 84	6 : 85
**DD-7**	36.37 ± 7.46	36.21 ± 7.29	35	35	0 : 100	6 : 86
**BI**	38.42 ± 8.58	38.23 ± 8.49	35	35	0 : 90	7 : 82
**CD**	37.86 ± 8.32	37.69 ± 8.22	35	35	3 : 85	4 : 84

Supplementary Table 3 – Sediments specific reads. Reads of sediment sections homologous to reads from the overlying water column and brine layers' datasets were eliminated.

Sample	Reads	Subtracted reads	Sediment Specific reads	Sediment Specific reads (%)
**ATIID-1a**	388642	223618	165024	42%
**ATIID-1b**	390240	277000	113240	29%
**ATIID-2**	81388	27645	53743	66%
**ATIID-3**	209973	72287	137686	66%
**ATIID-4**	57447	19305	38142	66%
**ATIID-5**	124488	41897	82591	66%
**ATIID-6**	88058	33688	54370	62%
**DD-1**	240352	75334	165018	69%
**DD-2**	101602	38791	62811	62%
**DD-3**	66033	27130	38903	59%
**DD-4**	129184	52320	76864	59%
**DD-5**	157017	67748	89269	57%
**DD-6**	182942	77111	105831	58%
**DD-7**	780210	276952	503258	65%
**BI**	104043	35151	68892	66%
**CD**	124159	43598	80561	65%

Supplementary Table 4: ATIID-1 group specific taxa.

Taxonomy	ATIID-1: mean rel. freq. (%)	ATIID-1: std. dev. (%)	<All other samples>: mean rel. freq. (%)	<All other samples>: std. dev. (%)	p-values	p-values (corrected)	Difference between means	95.0% lower CI	95.0% upper CI
**Archaea|Euryarchaeota|unclassified Euryarchaeota|Candidatus Parvarchaeum**	0.007307	0.000641	0	0	0.001048	0.023885	0.007307	0.006666	0.007948
**Archaea|Nanoarchaeota|Nanoarchaeum|Nanoarchaeum equitans|Nanoarchaeum equitans Kin4-M**	0.00477	0.000528	0	0	0.001954	0.031268	0.00477	0.004242	0.005298
**Archaea|Thaumarchaeota|Nitrosopumilales|Nitrosopumilaceae|Candidatus Nitrosoarchaeum|Candidatus Nitrosoarchaeum koreensis|Candidatus Nitrosoarchaeum koreensis MY1**	0.006259	0.000805	0	0	0.002787	0.040067	0.006259	0.005454	0.007065
**Bacteria|Actinobacteria phylum|Actinobacteria|Actinobacteridae|Actinomycetales|Corynebacterineae|Gordoniaceae|Gordonia**	0.006865	0.000199	0.00033	0.000853	0.000332	0.016279	0.006535	0.005989	0.007065
**Bacteria|Actinobacteria phylum|Actinobacteria|Actinobacteridae|Actinomycetales|Corynebacterineae|Nocardiaceae|Rhodococcus**	0.009125	0.001177	9.94E-05	0.000358	0.002859	0.040563	0.009025	0.007749	0.010302
**Bacteria|Actinobacteria phylum|Actinobacteria|Actinobacteridae|Actinomycetales|Micrococcineae|Beutenbergiaceae|Beutenbergia|Beutenbergia cavernae|Beutenbergia cavernae DSM 12333**	0.004329	8.68E-05	0	0	4.25E-05	0.014327	0.004329	0.004242	0.004415
**Bacteria|Actinobacteria phylum|Actinobacteria|Actinobacteridae|Actinomycetales|Streptomycineae|Streptomycetaceae|Streptomyces|Streptomyces viridochromogenes|Streptomyces viridochromogenes DSM 40736**	0.004329	8.68E-05	7.1E-05	0.000256	6.35E-05	0.014327	0.004258	0.004045	0.004415
**Bacteria|Actinobacteria phylum|Actinobacteria|Actinobacteridae|Actinomycetales|Streptosporangineae|Streptosporangiaceae|Streptosporangium|Streptosporangium roseum|Streptosporangium roseum DSM 43021**	0.005679	0.000381	0	0	0.000611	0.018886	0.005679	0.005298	0.00606
**Bacteria|Actinobacteria phylum|Actinobacteria|Rubrobacteridae|Rubrobacterales|Rubrobacterineae|Rubrobacteraceae|Rubrobacter|Rubrobacter xylanophilus|Rubrobacter xylanophilus DSM 9941**	0.012406	0.000926	0	0	0.00074	0.020108	0.012406	0.01148	0.013331
**Bacteria|Aquificae phylum|Aquificae|Aquificales|Aquificaceae|Hydrogenobacter|Hydrogenobacter thermophilus|Hydrogenobacter thermophilus TK-6**	0.006121	6.09E-05	0.000589	0.001557	0.000856	0.021707	0.005531	0.004598	0.006182
**Bacteria|Chlamydiae/Verrucomicrobia group|Chlamydiae|Chlamydiia|Chlamydiales|Waddliaceae|Waddlia|Waddlia chondrophila**	0.005073	0.000225	2.84E-05	0.000102	0.000294	0.016146	0.005045	0.004791	0.005298
**Bacteria|Chlamydiae/Verrucomicrobia group|Verrucomicrobia|Opitutae|Opitutales|Opitutaceae|unclassified Opitutaceae|Opitutaceae bacterium TAV5**	0.011964	0.001367	0.000907	0.001869	0.002934	0.040563	0.011057	0.009094	0.012985
**Bacteria|Chloroflexi phylum|Anaerolineae|Anaerolineales|Anaerolineaceae|Anaerolinea|Anaerolinea thermophila|Anaerolinea thermophila UNI-1**	0.01715	0.001395	0	0	0.00091	0.022561	0.01715	0.015755	0.018545
**Bacteria|Chloroflexi phylum|Chloroflexi|Chloroflexales|Chloroflexaceae|Roseiflexus**	0.018085	0.001306	9.94E-05	0.000358	0.000688	0.019329	0.017985	0.01658	0.019391
**Bacteria|Chloroflexi phylum|Dehalococcoidetes|Dehalococcoidales|Dehalococcoidaceae|Dehalococcoides|Dehalococcoides mccartyi|Dehalococcoides sp. CBDB1**	0.009237	0.00136	0	0	0.004077	0.048897	0.009237	0.007878	0.010597
**Bacteria|Chloroflexi phylum|Dehalococcoidetes|Dehalogenimonas|Dehalogenimonas lykanthroporepellens|Dehalogenimonas lykanthroporepellens BL-DC-9**	0.015825	7.01E-05	0	0	6.54E-06	0.012801	0.015825	0.015755	0.015895
**Bacteria|Chloroflexi phylum|environmental samples Green non-sulfur bacteria|uncultured Chloroflexi bacterium HF0500 03M05**	0.013315	0.001835	0	0	0.003392	0.045567	0.013315	0.01148	0.015149
**Bacteria|Chloroflexi phylum|Thermomicrobia|Sphaerobacteridae|Sphaerobacterales|Sphaerobacterineae|Sphaerobacteraceae|Sphaerobacter|Sphaerobacter thermophilus|Sphaerobacter thermophilus DSM 20745**	0.035979	0.000227	8.52E-05	0.000307	1.18E-05	0.012801	0.035894	0.035582	0.036206
**Bacteria|Chloroflexi phylum|Thermomicrobia|Thermomicrobiales|Thermomicrobiaceae|Thermomicrobium|Thermomicrobium roseum|Thermomicrobium roseum DSM 5159**	0.013263	0.001749	0	0	0.002919	0.040563	0.013263	0.011513	0.015012

**Bacteria|Cyanobacteria|Chroococcales|Acaryochloris**	0.006121	6.09E-05	0.000857	0.00268	0.003766	0.048023	0.005264	0.003724	0.006121
**Bacteria|Cyanobacteria|Chroococcales|Synechococcus|Synechococcus sp. PCC 7335**	0.006865	0.000199	0.000558	0.00201	0.001203	0.024679	0.006308	0.005192	0.007065
**Bacteria|Fibrobacteres/Acidobacteria group|Acidobacteria|environmental samples Acidobacteria|uncultured Acidobacteria bacterium**	0.008354	0.000477	0	0	0.000459	0.016939	0.008354	0.007878	0.008831
**Bacteria|Fibrobacteres/Acidobacteria group|Acidobacteria|Solibacteres|Solibacterales|Solibacteraceae|Candidatus Solibacter|Candidatus Solibacter usitatus|Candidatus Solibacter usitatus Ellin6076**	0.064175	0.008541	0.002148	0.002968	0.003444	0.045996	0.062027	0.052509	0.07159
**Bacteria|Fibrobacteres/Acidobacteria group|Acidobacteria|unclassified Acidobacteria|Candidatus Koribacter|Candidatus Koribacter versatilis|Candidatus Koribacter versatilis Ellin345**	0.017946	0.002051	0.000156	0.000563	0.002143	0.032893	0.01779	0.015583	0.019997
**Bacteria|Firmicutes|Bacilli|Bacillales|Alicyclobacillaceae|Alicyclobacillus|Alicyclobacillus acidocaldarius|Alicyclobacillus acidocaldarius LAA1**	0.005073	0.000225	0	0	0.000253	0.015003	0.005073	0.004848	0.005298
**Bacteria|Firmicutes|Bacilli|Bacillales|Bacillaceae|Bacillus|Bacillus cereus group|Bacillus cereus|Bacillus cereus R309803**	0.005073	0.000225	0	0	0.000253	0.015003	0.005073	0.004848	0.005298
**Bacteria|Firmicutes|Bacilli|Bacillales|Paenibacillaceae|Paenibacillus|Paenibacillus mucilaginosus**	0.004935	0.000519	0	0	0.001678	0.028689	0.004935	0.004415	0.005454
**Bacteria|Firmicutes|Bacilli|Bacillales|Planococcaceae**	0.004026	0.00039	0	0	0.001293	0.02516	0.004026	0.003636	0.004415
**Bacteria|Firmicutes|Clostridia|Clostridiales|Clostridiales incertae sedis|Clostridiales Family XVII. Incertae Sedis|Thermaerobacter**	0.007774	0.000709	8.52E-05	0.000307	0.001187	0.02458	0.007689	0.006894	0.008484
**Bacteria|Firmicutes|Clostridia|Clostridiales|Eubacteriaceae**	0.004026	0.00039	0.000274	0.000933	0.002622	0.038431	0.003752	0.002974	0.004401
**Bacteria|Firmicutes|Clostridia|Thermoanaerobacterales|Thermoanaerobacterales Family IV. Incertae Sedis|Mahella|Mahella australiensis|Mahella australiensis 50-1 BON**	0.00703	0.000848	0.000373	0.000992	0.003003	0.041078	0.006657	0.005436	0.007878
**Bacteria|Gemmatimonadetes|Gemmatimonadetes class|Gemmatimonadales|Gemmatimonadaceae|Gemmatimonas|Gemmatimonas aurantiaca|Gemmatimonas aurantiaca T-27**	0.017782	0.001003	0.000696	0.002066	0.000628	0.018886	0.017085	0.015386	0.018785
**Bacteria|Nitrospirae|Nitrospira class|Nitrospirales|Nitrospiraceae|Thermodesulfovibrio|Thermodesulfovibrio yellowstonii|Thermodesulfovibrio yellowstonii DSM 11347**	0.005818	0.000364	0	0	0.000512	0.017792	0.005818	0.005454	0.006182
**Bacteria|Proteobacteria|Alphaproteobacteria|Rhizobiales|Bradyrhizobiaceae|Bradyrhizobium|Bradyrhizobium sp. BTAi1**	0.004935	0.000519	7.1E-05	0.000256	0.001776	0.029868	0.004864	0.004273	0.005454
**Bacteria|Proteobacteria|Alphaproteobacteria|Rhizobiales|Bradyrhizobiaceae|unclassified Bradyrhizobiaceae|Bradyrhizobiaceae bacterium SG-6C**	0.005679	0.000381	0	0	0.000611	0.018886	0.005679	0.005298	0.00606
**Bacteria|Proteobacteria|Alphaproteobacteria|Rhizobiales|Hyphomicrobiaceae|Hyphomicrobium|Hyphomicrobium sp. MC1**	0.004026	0.00039	0	0	0.001293	0.02516	0.004026	0.003636	0.004415
**Bacteria|Proteobacteria|Alphaproteobacteria|Rhizobiales|Xanthobacteraceae|Starkeya|Starkeya novella|Starkeya novella DSM 506**	0.005982	0.000684	0	0	0.002082	0.032637	0.005982	0.005298	0.006666
**Bacteria|Proteobacteria|Betaproteobacteria|Burkholderiales|Alcaligenaceae|Bordetella**	0.005679	0.000381	0.000128	0.000461	0.000683	0.019329	0.005551	0.005043	0.00606
**Bacteria|Proteobacteria|Betaproteobacteria|Burkholderiales|Comamonadaceae|Variovorax|Variovorax paradoxus|Variovorax paradoxus S110**	0.004026	0.00039	0.000473	0.001164	0.003815	0.048051	0.003553	0.002691	0.004415
**Bacteria|Proteobacteria|Betaproteobacteria|Rhodocyclales|Rhodocyclaceae|Methyloversatilis|Methyloversatilis universalis|Methyloversatilis universalis FAM5**	0.004026	0.00039	0	0	0.001293	0.02516	0.004026	0.003636	0.004415
**Bacteria|Proteobacteria|delta/epsilon subdivisions|Deltaproteobacteria|Desulfobacterales|Desulfobulbaceae**	0.004329	8.68E-05	0.000274	0.000933	0.000623	0.018886	0.004055	0.003449	0.004415
**Bacteria|Proteobacteria|delta/epsilon subdivisions|Deltaproteobacteria|Desulfovibrionales|Desulfovibrionaceae|Desulfovibrio|Desulfovibrio alaskensis|Desulfovibrio alaskensis G20**	0.00477	0.000528	0	0	0.001954	0.031268	0.00477	0.004242	0.005298
**Bacteria|Proteobacteria|delta/epsilon subdivisions|Deltaproteobacteria|Desulfovibrionales|Desulfovibrionaceae|Desulfovibrio|Desulfovibrio salexigens|Desulfovibrio salexigens DSM 2638**	0.004026	0.00039	9.94E-05	0.000358	0.001579	0.028274	0.003926	0.003437	0.004415
**Bacteria|Proteobacteria|delta/epsilon subdivisions|Deltaproteobacteria|environmental samples delta subdivision**	0.014336	0.000207	0.007896	0.003247	0.003899	0.048066	0.00644	0.004872	0.008224

Bacteria|Proteobacteria|Gammaproteobacteria|Alteromonadales|Idiomarinaceae|Idiomarina	0.056843	0.00739	0.004292	0.003781	0.00379	0.048051	0.052551	0.043713	0.061071
Bacteria|Proteobacteria|Gammaproteobacteria|Alteromonadales|Shewanellaceae|Shewanella|Shewanella putrefaciens	0.002537	0.000113	0	0	0.000253	0.015003	0.002537	0.002424	0.002649
Bacteria|Proteobacteria|Gammaproteobacteria|Chromatiales|Ectothiorhodospiraceae	0.010614	0.0009	0.001643	0.003182	0.003626	0.047676	0.008971	0.006751	0.010951
Bacteria|Proteobacteria|Gammaproteobacteria|Oceanospirillales|Oceanospirillaceae|Oceanospirillum	0.007471	0.000407	7.1E-05	0.000256	0.00045	0.016939	0.0074	0.006923	0.007878
Bacteria|Proteobacteria|Gammaproteobacteria|Pseudomonadales|Pseudomonadaceae|Pseudomonas|Pseudomonas aeruginosa group|Pseudomonas aeruginosa	0.004935	0.000519	0	0	0.001678	0.028689	0.004935	0.004415	0.005454
Bacteria|Proteobacteria|Gammaproteobacteria|Pseudomonadales|Pseudomonadaceae|Pseudomonas|Pseudomonas fluorescens group	0.067076	0.002611	0.0041	0.003505	0.000247	0.015003	0.062976	0.059262	0.066834
Bacteria|Proteobacteria|Gammaproteobacteria|Thiotrichales|Piscirickettsiaceae|Methylophaga|Methylophaga aminisulfidivorans|Methylophaga aminisulfidivorans MP	0.004632	0.000216	0	0	0.000315	0.016146	0.004632	0.004415	0.004848
Bacteria|Proteobacteria|Gammaproteobacteria|unclassified Gammaproteobacteria|sulfur-oxidizing symbionts	0.008657	0.000174	0.000674	0.001947	0.000672	0.019329	0.007983	0.006888	0.008831
Bacteria|Spirochaetes|Spirochaetia|Spirochaetales|Brachyspiraceae|Brachyspira	0.005073	0.000225	0.000128	0.000461	0.0004	0.016352	0.004945	0.004592	0.005298
Bacteria|Spirochaetes|Spirochaetia|Spirochaetales|Leptospiraceae|Leptospira	0.019712	0.000285	0.004052	0.004819	0.001029	0.023692	0.015661	0.013005	0.017971
Bacteria|Spirochaetes|Spirochaetia|Spirochaetales|Spirochaetaceae|Treponema	0.00954	0.001057	0.001382	0.001951	0.00404	0.048756	0.008158	0.006433	0.009915
Bacteria|Thermotogae phylum|Thermotogae|Thermotogales|Thermotogaceae|Thermotoga	0.005376	7.76E-05	0	0	2.55E-05	0.014327	0.005376	0.005298	0.005454
Bacteria|unclassified Bacteria|Caldithrix|Caldithrix abyssi|Caldithrix abyssi DSM 13497	0.06305	0.003001	0.025461	0.009196	0.001659	0.028689	0.037589	0.030528	0.043899
Bacteria|unclassified Bacteria|candidate division OP1|environmental samples candidate division OP1|uncultured candidate division OP1 bacterium	0.012709	0.001229	0.000805	0.001972	0.002031	0.032256	0.011904	0.00987	0.013537
Bacteria|unclassified Bacteria|candidate division TM7	0.011964	0.001367	0.000599	0.001313	0.002617	0.038431	0.011366	0.009494	0.013189
Bacteria|unclassified Bacteria|Poribacteria|Candidatus Poribacteria sp. WGA-A3	0.123582	0.013295	0.002257	0.003638	0.001923	0.031219	0.121325	0.106698	0.135775
Bacteria|unclassified Bacteria|Thermobaculum|Thermobaculum terrenum|Thermobaculum terrenum ATCC BAA-798	0.017176	0.000397	0	0	5.3E-05	0.014327	0.017176	0.016779	0.017573
unclassified sequences|environmental samples unclassified|prokaryotic environmental samples|uncultured marine microorganism HF4000 001A02	0.008051	0.00078	0.000952	0.002268	0.003735	0.048023	0.0071	0.005405	0.008614
unclassified sequences|environmental samples unclassified|prokaryotic environmental samples|uncultured marine microorganism HF4000 ANIW93N21	0.020457	0.000146	0	0	1.7E-05	0.012801	0.020457	0.020311	0.020603
unclassified sequences|environmental samples unclassified|prokaryotic environmental samples|uncultured marine microorganism HF4000 APKG10K24	0.014475	0.000537	0	0	0.0002	0.015003	0.014475	0.013937	0.015012
unclassified sequences|environmental samples unclassified|prokaryotic environmental samples|uncultured marine microorganism HF4000 APKG7N23	0.005679	0.000381	0.000838	0.001845	0.002912	0.040563	0.004841	0.003623	0.005804
unclassified sequences|environmental samples unclassified|prokaryotic environmental samples|uncultured marine microorganism HF4000 APKG8C21	0.014362	0.001999	0	0	0.0035	0.046464	0.014362	0.012363	0.016361
unclassified sequences|environmental samples unclassified|uncultured organism	0.022526	0.001317	0	0	0.00048	0.016939	0.022526	0.021209	0.023843
Viruses|ssDNA viruses|Nanoviridae	0.005073	0.000225	0	0	0.000253	0.015003	0.005073	0.004848	0.005298

Supplementary Table 5 – Sediment subsections comparison using Shannon Diversity and Simpson Reciprocal indices.

		ATIID							DD								
**Phylogenetic Approach**	**Section Name**	**1a**	**1b**	**2**	**3**	**4**	**5**	**6**	**1**	**2**	**3**	**4**	**5**	**6**	**7**	**BI**	**CD**
**v6v4 16S rRNA pyrotags (archaea)**	**Richness *R*** [1]	16	14	17	13	14	20	22	38	18	23	22	22	22	22	22	20
	**Shannon Entropy *H*** [2]	1.57	0.37	0.34	0.25	0.21	0.86	0.79	1.44	0.87	0.63	0.94	0.91	0.91	0.70	0.85	0.68
	**Evenness *E*** [3]	0.57	0.14	0.12	0.10	0.08	0.29	0.26	0.40	0.30	0.20	0.30	0.29	0.29	0.23	0.28	0.23
	**Simpson Index *D*** [4]	0.31	0.87	0.88	0.91	0.92	0.59	0.63	0.32	0.59	0.74	0.56	0.58	0.58	0.70	0.61	0.71
	**Simpson Reciprocal Index *1/D***	3.23	1.16	1.14	1.10	1.08	1.69	1.57	3.16	1.70	1.35	1.78	1.73	1.72	1.43	1.64	1.41
**v6v4 16S rRNA pyrotags (bacteria)**	**Richness *R*** [1]	122	103	98	93	98	145	175	220	157	154	158	166	146	160	159	134
	**Shannon Entropy *H*** [2]	2.60	2.57	2.52	2.52	2.59	2.84	2.88	3.38	2.90	2.84	2.91	2.90	2.89	2.85	2.92	2.88
	**Evenness *E*** [3]	0.54	0.55	0.55	0.56	0.57	0.57	0.56	0.63	0.57	0.56	0.57	0.57	0.58	0.56	0.58	0.59
	**Simpson Index *D*** [4]	0.14	0.16	0.15	0.16	0.15	0.10	0.10	0.07	0.10	0.10	0.09	0.10	0.10	0.10	0.09	0.09
	**Simpson Reciprocal Index *1/D***	7.39	6.38	6.47	6.22	6.77	9.94	10.13	14.21	10.04	9.70	10.60	10.42	10.31	9.81	10.54	10.82
**Protein based phylogeny of metagenomic reads (archaea)**	**Richness *R*** [1]	228	214	44	97	33	60	64	63	56	61	98	130	126	110	61	63
	**Shannon Entropy *H*** [2]	3.96	3.77	2.87	3.20	2.71	3.03	3.26	2.66	2.92	3.25	3.57	3.75	3.62	3.04	3.07	3.03
	**Evenness *E*** [3]	0.73	0.70	0.76	0.70	0.77	0.74	0.78	0.64	0.73	0.79	0.78	0.77	0.75	0.65	0.75	0.73
	**Simpson Index *D*** [4]	0.06	0.08	0.14	0.14	0.14	0.13	0.09	0.22	0.15	0.08	0.07	0.07	0.08	0.16	0.14	0.14
	**Simpson Reciprocal Index *1/D***	17.13	13.21	7.00	7.25	7.27	7.70	11.40	4.54	6.65	11.77	13.98	14.19	11.81	6.38	7.40	7.35
**Protein based phylogeny of metagenomic reads (bacteria)**	**Richness *R*** [1]	1305	1074	341	659	294	465	329	597	402	290	478	567	631	1029	408	439
	**Shannon Entropy *H*** [2]	4.76	5.03	4.04	4.74	4.46	4.59	4.30	4.52	4.33	4.38	4.68	4.85	4.92	4.73	4.58	4.50
	**Evenness *E*** [3]	0.66	0.72	0.69	0.73	0.78	0.75	0.74	0.71	0.72	0.77	0.76	0.76	0.76	0.68	0.76	0.74
	**Simpson Index *D*** [4]	0.04	0.03	0.06	0.04	0.04	0.04	0.06	0.06	0.05	0.05	0.04	0.03	0.03	0.04	0.04	0.05
	**Simpson Reciprocal Index *1/D***	24.93	30.88	18.03	23.73	25.11	22.89	18.13	16.77	21.59	21.08	24.49	29.10	30.80	23.81	23.65	20.09

**Protein based phylogeny of metagenomic reads**	**Richness *R*** [1]	1883	1588	580	1069	507	777	602	969	690	545	845	993	1086	1550	713	767
	**Shannon Entropy *H*** [2]	2.14	1.62	2.25	2.34	2.29	2.29	2.10	2.21	2.31	2.02	2.16	2.21	2.24	2.22	2.18	2.21
	**Evenness *E*** [3]	0.28	0.22	0.35	0.34	0.37	0.34	0.33	0.32	0.35	0.32	0.32	0.32	0.32	0.30	0.33	0.33
	**Simpson Index *D*** [4]	0.45	0.59	0.41	0.39	0.40	0.40	0.45	0.40	0.39	0.47	0.45	0.44	0.43	0.41	0.43	0.42
	**Simpson Reciprocal Index *1/D***	2.24	1.70	2.47	2.56	2.52	2.50	2.24	2.51	2.54	2.13	2.24	2.29	2.34	2.46	2.32	2.36

1: Total number of species in the community.

2: $H = - \sum p_i \ln (p_i)$, where pi is the proportion of the ith species from the total number of individuals in all species. *H* measures the rarity and commonness of species in a community.

3: $E = H / \ln(R)$.

4: $D = \sum p_i^2$. *D* measures the probability that two individuals randomly selected from a sample will belong to the same species.

Supplementary Table 6 – Correlation of bacterial and viral abundances to the abundances of mobile genetic elements.

	Bacteria *vs.* Plasmid	Bacteria *vs.* Integron	Bacteria *vs.* IS	Viruses *vs.* Plasmid	Viruses *vs.* Integron	Viruses *vs.* IS
**Pearson r**						
**r**	0.6029	0.7904	0.8874	-0.7162	-0.9058	-0.7545
**P value**						
**P (two-tailed)**	0.0134	0.0003	< 0.0001	0.0018	< 0.0001	0.0007
**P value summary**	*	***	****	**	****	***
**Significant (alpha = 0.05)**	Yes	Yes	Yes	Yes	Yes	Yes
